# Accuracy and Precision of Three Consumer-Grade Motion Sensors During Overground and Treadmill Walking in People With Parkinson Disease: Cross-Sectional Comparative Study

**DOI:** 10.2196/14059

**Published:** 2020-01-16

**Authors:** Byron Lai, Jeffer E Sasaki, Brenda Jeng, Katie L Cederberg, Marcas M Bamman, Robert W Motl

**Affiliations:** 1 Department of Physical Therapy University of Alabama at Birmingham Birmingham, AL United States; 2 Federal University of Triangulo Mineiro Uberaba Brazil; 3 UAB Center for Exercise Medicine University of Alabama at Birmingham Birmingham, AL United States; 4 Department of Cell, Developmental, and Integrative Biology University of Alabama at Birmingham Birmingham, AL United States

**Keywords:** wearable electronic devices, wearable, fitness tracker, accelerometer, reproducibility, Parkinson disease, disabled persons, exercise

## Abstract

**Background:**

Wearable motion sensors are gaining popularity for monitoring free-living physical activity among people with Parkinson disease (PD), but more evidence supporting the accuracy and precision of motion sensors for capturing step counts is required in people with PD.

**Objective:**

This study aimed to examine the accuracy and precision of 3 common consumer-grade motion sensors for measuring actual steps taken during prolonged periods of overground and treadmill walking in people with PD.

**Methods:**

A total of 31 ambulatory participants with PD underwent 6-min bouts of overground and treadmill walking at a comfortable speed. Participants wore 3 devices (Garmin Vivosmart 3, Fitbit One, and Fitbit Charge 2 HR), and a single researcher manually counted the actual steps taken. Accuracy and precision were based on absolute and relative metrics, including intraclass correlation coefficients (ICCs) and Bland-Altman plots.

**Results:**

Participants walked 628 steps over ground based on manual counting, and Garmin Vivosmart, Fitbit One, and Fitbit Charge 2 HR devices had absolute (relative) error values of 6 (6/628, 1.0%), 8 (8/628, 1.3%), and 30 (30/628, 4.8%) steps, respectively. ICC values demonstrated excellent agreement between manually counted steps and steps counted by both Garmin Vivosmart (0.97) and Fitbit One (0.98) but poor agreement for Fitbit Charge 2 HR (0.47). The absolute (relative) precision values for Garmin Vivosmart, Fitbit One, and Fitbit Charge 2 HR were 11.1 (11.1/625, 1.8%), 14.7 (14.7/620, 2.4%), and 74.4 (74.4/598, 12.4%) steps, respectively. ICC confidence intervals demonstrated low variability for Garmin Vivosmart (0.96 to 0.99) and Fitbit One (0.93 to 0.99) but high variability for Fitbit Charge 2 HR (–0.57 to 0.74). The Fitbit One device maintained high accuracy and precision values for treadmill walking, but both Garmin Vivosmart and Fitbit Charge 2 HR (the wrist-worn devices) had worse accuracy and precision for treadmill walking.

**Conclusions:**

The waist-worn sensor (Fitbit One) was accurate and precise in measuring steps with overground and treadmill walking. The wrist-worn sensors were accurate and precise only during overground walking. Similar research should inform the application of these devices in clinical research and practice involving patients with PD.

## Introduction

### Background

Wearable motion sensors have been applied for monitoring and promoting free-living ambulatory physical activity based on the outcome of steps taken per unit time among people with Parkinson disease (PD) [1]. Such applications, nevertheless, require evidence supporting the accuracy and precision of the motion sensors for capturing actual steps taken as a metric of ambulation.

We located 2 studies that have examined the accuracy and precision of motion sensors for capturing steps during relatively short 2-min periods of overground walking in patients with PD [2,3]. Assessment during such a short bout of walking does not provide an accurate and precise measurement associated with the energy systems required for free-living, ambulatory physical activity in neurological diseases [4]. The study of motion sensor precision, in particular, requires longer bouts of walking, as step count recordings from motion sensors can be compromised over time by subtle gait disturbances [5] brought about by energetic fatigue that may occur in PD and other neurological diseases. Furthermore, there is a need to determine the accuracy and precision of motion sensors during treadmill walking, as this modality is often prescribed for gait training and physical activity in PD.

### Objectives

This study examined the accuracy and precision of 3 common motion sensors (Garmin Vivosmart 3, Fitbit One, and Fitbit Charge 2 HR) for measuring actual steps taken during longer periods of overground and treadmill walking in people with PD.

## Methods

### Participants

Community-dwelling participants were recruited from local clinics, support groups, and community events. Inclusion criteria were (1) neurologist-confirmed diagnosis of idiopathic PD (presence of bradykinesia plus rigidity and resting tremor), (2) age between 50 and 74 years, (3) physically independent with bilateral symptoms indicative of a Hoehn and Yahr stage 2 or 3 (mild-to-moderate disability) that was confirmed by a neurologist and self-reported by the participant, and (4) ability to walk for 6 min (without an assistive device). Exclusion criteria were (1) motor symptoms because of neuroleptic medication or a stroke, (2) any condition that prevented the participant from being able to follow the protocol or participate safely, and (3) not responsive to dopaminergic medications. Written informed consent was obtained from all participants, and the University Institutional Review Board approved the protocol. The study was conducted in accordance with the principles of ethical human research as defined in the Declaration of Helsinki.

### Motion Sensors

We examined the accuracy of 3 consumer-grade motion sensors: Garmin Vivosmart 3 (Garmin), Fitbit One (Fitbit Inc), and Fitbit Charge 2 HR (Fitbit Inc). The devices were worn on the less affected side: the 2 wrist-worn devices (Garmin Vivosmart 3 and Fitbit Charge 2 HR) on the less affected arm and the waist-worn sensor (Fitbit One) on the side of the less affected leg. We chose these monitors based on popularity, availability, and application in the general adult population [5] and people with neurological diseases [6], although Fitbit One is now no longer commercially available.

### Overground Protocol

Participants completed one 6-min bout of overground walking around an indoor, oval track marked with cones. Participants were instructed to walk at a comfortable walking speed (CWS) that resembled walking speed undertaken during normal daily activities. The single speed was chosen because people with PD typically reach an average of 64% of peak oxygen consumption while walking at a self-selected treadmill speed and might undergo this speed for treadmill training [7]. Research staff recorded the step count values from the motion sensors immediately before and after the walking bout. One researcher manually recorded the steps taken using a handheld tally counter (ie, direct observation as a gold standard). This researcher underwent 3 months of training for proficiency with a high degree of accuracy, and this was the researcher’s only responsibility during the walking trials. Furthermore, we noted that this training and procedure produces accurate data in our laboratory and focused on participant safety during the study with a minimal amount of study staff available during a test session. The distance participants walked was recorded for determining the CWS for the subsequent treadmill protocol.

### Treadmill Protocol

Participants undertook 6 min of walking on a motor-driven treadmill (Trackmaster TMX428, Full Vision). The speed was determined as the CWS from the overground trial. We selected this speed for comparability of accuracy and precision with the overground bout of walking and further recognize that the metabolic demand of CWS corresponds with an intensity of 64% of peak oxygen consumption and is consistent with training zones recommended for PD [7]. The protocols for recording manually counted and device-recorded steps matched the overground protocol.

### Procedure

Participants completed the study in a single visit. Participants provided demographic, anthropometric, and clinical information and then completed the Physical Activity Readiness Questionnaire for identifying contraindications for engaging in physical activity. The stage of PD was measured by using the Hoehn and Yahr scale. Motor symptoms were captured via the Movement Disorder Society version of the Motor Examination of the Unified Parkinson’s Disease Rating Scale (MDS-UPDRS-III); both were administered by 2 research staff who completed the MDS-UPDRS training. This was followed by the overground and then treadmill bouts of walking; there was 5 min of rest between bouts. Participants were compensated US $25 upon completing the study.

### Data Analysis

Accuracy and precision were based on absolute and relative metrics [6], including intraclass correlation coefficients (ICCs2,1) and Bland-Altman plots. Absolute accuracy was measured by the mean difference between device and manually recorded steps. Metrics for relative accuracy included (1) mean percentage error, (2) frequency of large errors, and (3) ICCs using IBM SPSS Statistics version 24 (IBM Corp). The mean percentage error was expressed as the difference between actual (manually counted steps) and observed steps, divided by the actual steps, and multiplied by 100. The frequency of cases for errors was categorized into 1 of 3 categories per device: ≥5%, ≥10%, and ≥25% [6]. The ICCs demonstrated the degree of agreement between manual and device-recorded steps. ICC values were interpreted as follows: less than 0.5=poor, 0.5 to 0.75=moderate, 0.75 to 0.9=good, and greater than 0.9=excellent agreement [8].

Absolute precision was based on the standard deviation of the mean difference between device and manually recorded steps. Relative precision was expressed as the coefficient of variation between device and manually recorded steps and ICC confidence intervals (the strength of agreement between manual and device-recorded steps over repeated measures).

**Figure 1:**
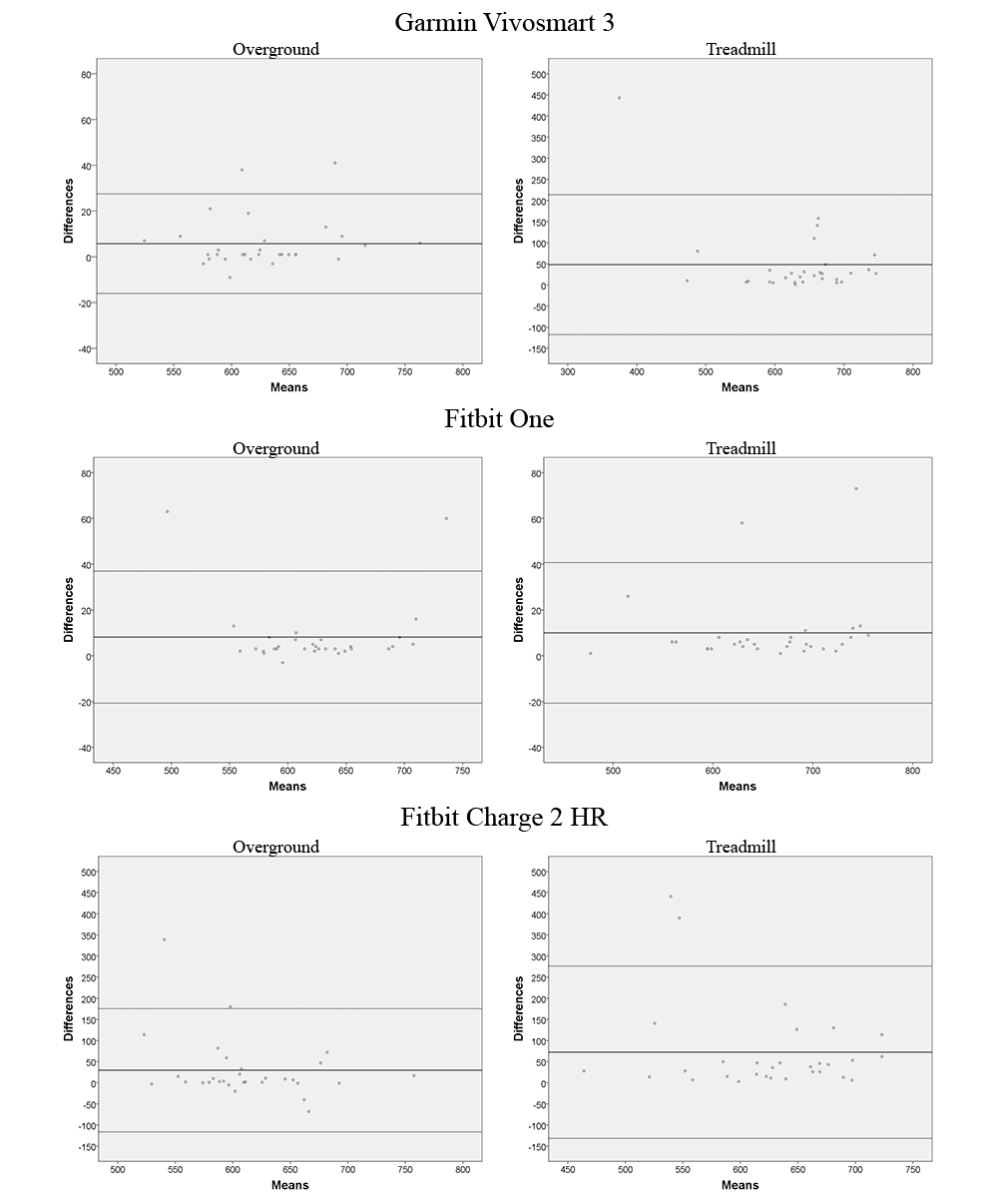
Bland-Altman plots for each motion sensor and walking condition.

Bland-Altman plots were produced as visual representations of accuracy and precision. Bland-Altman plots represent the difference between manually recorded steps and device-recorded steps against the mean of the 2 methods. As presented in Figure 1, the solid line represents the mean difference between manually counted steps and those obtained from the device (absolute accuracy). The limits of agreement were set at 95%, as represented by the 2 dotted lines (relative precision).

## Results

### Participants

We contacted and screened 71 potential participants, and 38 of them satisfied eligibility criteria. Of those persons, 31 enrolled in and completed the study and were included for analysis; 7 persons declined the invitation for participation. The characteristics of the participants are presented in Table 1. The mean age of diagnosis and representation of males and females were comparable with US prevalence estimates [9]. Moreover, 26 participants had a Hoehn and Yahr score of 2, and 5 participants had a Hoehn and Yahr score of 3. In addition, 16 participants had gait impairments (9=slight and 7=mild), 13 participants had postural deviations (10=mild and 3=moderate), 8 participants had slight freezing of gait (this did not occur during either the overground or treadmill walking trials), and 1 participant had only minor bilateral gait impairment.

**Table 1:** Demographic and clinical characteristics (n=31).

Characteristics	Mean (SD)	Range
Age (years)	64.3 (6.3)	53-74
Height (cm)	170.3 (8.6)	155-188
Weight (kg)	79.7 (16.5)	54.8-121.5
Body mass index (kg/m^2^)	27.5 (4.9)	18.2-38.8
Years postdiagnosis	6.5 (5.2)	1-23
Movement Disorder Society version of the Motor Examination of the Unified Parkinson’s Disease Rating Scale	24 (1)	1-71
Walking speed (m/s)	1.05 (0.16)	0.6-1.3

Data collection was conducted between March 2018 and October 2018. Data from all 31 participants were analyzed, except for 2 trials where a device error occurred. There were no steps recorded in 1 case likely caused by Wi-Fi internet instability while syncing the device with the Android tablet, and the other case resulted from a device that had insufficient battery power.

### Accuracy

The absolute and relative metrics of accuracy per device and walking condition are presented in Table 2. Participants walked 628 steps overground based on manual counting, and the devices, Garmin Vivosmart, Fitbit One, and Fitbit Charge 2 HR, deviated from this manually counted value (absolute [relative] error values) by 6 (6/628, 1.0%), 8 (8/628, 1.3%), and 30 (30/628, 4.8%) steps, respectively. These values are visually represented by Bland-Altman plots in Figure 1. There were few cases of larger errors during overground walking for Garmin Vivosmart and Fitbit One, but Fitbit Charge 2 HR had more cases of larger errors (18/30, 60%). ICC values demonstrated excellent agreement between manually counted steps and both Garmin Vivosmart and Fitbit One but poor agreement for Fitbit Charge 2 HR.

Participants walked 660 steps on the treadmill based on manual counting, and the devices, Garmin Vivosmart, Fitbit One, and Fitbit Charge 2 HR, deviated from this value by 48 (48/660, 7.3%), 10 (10/660, 1.5%), and 72 (72/660, 10.9%) steps, respectively. Fitbit One had fewer cases of larger errors compared with Garmin Vivosmart and Fitbit Charge 2 HR. ICC values indicated that Garmin Vivosmart had moderate agreement, Fitbit One had excellent agreement, and Fitbit Charge 2 HR had poor agreement with manually recorded steps.

**Table 2:** Accuracy of motion sensors while walking at a comfortable speed.

Condition [actual steps, mean (95% CI)] and device			Absolute accuracy					Relative accuracy								
			Mean steps recorded (95% CI)		Mean difference in steps		Percentage error			n≥5% error		n≥10% error		n≥25% error		Intraclass correlation coefficient (2, 1)
**Overground,** **628 (609-647)**																
	Garmin Vivosmart 3 (n=30)	625 (606-644)		6		0.9			2		0		0		0.97	
	Fitbit One (n=31)	620 (600-639)		8		1.3			2		1		0		0.98	
	Fitbit Charge 2 HR (n=30)	598 (570-625)		30		4.4			10		6		2		0.47	
**Treadmill, 660 (633-686)**																
	Garmin Vivosmart 3 (n=30)	609 (568-649)		48		7.4			9		6		2		0.67	
	Fitbit One (n=31)	650 (624-675)		10		1.5			2		0		0		0.98	
	Fitbit Charge 2 HR (n=30)	587 (553-621)		72		10.3			16		7		3		0.27	

### Precision

The absolute and relative precision metrics per device and condition are provided in Table 3. With overground walking, the absolute and relative precision values (SD of mean difference [coefficient of variation]) for Garmin Vivosmart, Fitbit One, and Fitbit Charge 2 HR were 11.1 (11.1/625, 1.8%), 14.7 (14.7/620, 2.4%), and 74.4 (74.4/598, 12.4%), respectively. ICC confidence intervals for both Garmin Vivosmart and Fitbit One included narrow upper and lower limits that exceeded 0.9, indicating low variability and excellent agreement among most measures. Fitbit Charge 2 HR had higher variability with a confidence interval ranging from poor to moderate agreement.

**Table 3:** Precision of motion sensors while walking at a comfortable speed.

Condition [actual steps, mean (95% CI)] and devices			Absolute precision, mean difference SD		Relative precision	
				Coefficient of variation (%)		Intraclass correlation coefficient (2, 1) CI
**Overground,** **628 (609-647)**						
	Garmin Vivosmart 3 (n=30)	11.1		1.8		0.96 to 0.99
	Fitbit One (n=31)	14.7		2.4		0.93 to 0.99
	Fitbit Charge 2 HR (n=30)	74.4		12.4		–0.57 to 0.74
**Treadmill,** **660 (633-686)**						
	Garmin Vivosmart 3 (n=30)	84.5		13.9		0.27 to 0.85
	Fitbit One (n=31)	15.7		2.4		0.93 to 0.99
	Fitbit Charge 2 HR (n=30)	104		17.7		–0.26 to 0.61

Regarding treadmill walking, the absolute and relative precision values (SD of mean difference [coefficient of variation]) for Garmin Vivosmart, Fitbit One, and Fitbit Charge 2 HR were 84.5 (84.5/609, 13.9%), 15.7 (15.7/650, 2.4%), and 104 (104/587, 17.7%) steps, respectively. ICC confidence intervals demonstrated that Fitbit One had low variability and excellent agreement among most measures, whereas Garmin Vivosmart and Fitbit Charge 2 HR had higher variability as indicated by ICC confidence intervals of 0.27 to 0.85 and −0.26 to 0.61, respectively. This was supported by the Bland-Altman plots, demonstrating higher limits of agreement during treadmill walking compared with overground walking for both Garmin Vivosmart and Fitbit Charge 2 HR but not Fitbit One (Figure 1).

## Discussion

### Principal Findings

Study findings suggest that a waist-worn sensor (Fitbit One) can provide accurate and precise measurements of actual steps taken during overground or treadmill walking. These findings are consistent with previous treadmill walking research in the general adult population [10] and people with multiple sclerosis [6]. A waist-worn sensor, Fitbit One, can provide accurate and precise records of steps during both overground and treadmill walking. A wrist-worn sensor, Garmin Vivosmart 3, can provide accurate and precise records of step counts during overground walking in patients with PD but has noticeably worse estimates of step counts during treadmill walking. Fitbit Charge 2 HR provided poor estimates of step counts during both walking conditions.

The findings of this study suggest that wrist-worn devices provide noticeably worse measures of accuracy and precision during treadmill walking. One explanation for these findings is that 5 participants had difficulty with walking on a treadmill at a comfortable speed and intermittently used the handrails for support. After visual inspection of the outliers that were identified in the Bland-Altman plots during treadmill walking, 2 of the 3 largest errors in steps for both Garmin Vivosmart 3 and Fitbit Charge 2 HR were recorded in people who temporarily used handrails. Exclusion of these errors from the dataset would certainly lower the mean bias that was observed from the wrist-worn sensors, but this would not explain all the larger errors (>10% mean difference in steps) that were identified for each device. Handrail use was observed temporarily in 33% (2/6) of the larger errors recorded by Garmin Vivosmart 3 and 57% (4/7) of the larger errors recorded by Fitbit Charge 2 HR. No other observable trends were readily identified. Another possible explanation could be that waist-worn sensors are generally more accurate and precise than wrist-worn sensors [11].

Our results support and build on previous investigations of motion sensor accuracy and precision in PD. Regarding accuracy, mean percent errors and ICC values during overground walking for the Fitbit devices were similar with those reported of other Fitbit devices by Wendel et al (Fitbit Surge [wrist worn]: mean percent error=7.8 and ICC=0.38; Fitbit Zip [waist worn]: mean percent error=0.9 and ICC=0.98) [2]. Wendel et al [2] investigated the accuracy of 4 motion sensors (Fitbit Zip, Fitbit Surge, Jawbone Up Move, and Jawbone Up 2) for recording steps compared with manual counting, whereas people with PD underwent 4 trials of walking. Each trial lasted 2 min, and 1 trial was conducted at a CWS. The accuracy results of this study for Garmin Vivosmart 3 were also similar with those reported of Garmin Vivosmart HR by Lamont et al (mean percent error=2.7 and ICC=0.93) [3]. The researchers compared the accuracy of Fitbit Charge HR and Garmin Vivosmart for detecting steps from six 100-step walking trials at different cadences, and these readings were compared with those obtained from an accelerometer (ActivPAL3). However, Lamont et al [3] reported that a similar Fitbit motion sensor (Fitbit Charge HR) had an error rate of 2.8% and ICC of 0.88, which was lower than those reported in this study. A likely explanation for these findings is that participants with PD in this study had bilateral symptoms and a potentially higher amount of gait disturbances, which could have influenced step records over the longer 6-min walking period. Moreover, 44% of participants in the previous study were classified with a Hoehn and Yahr stage of 1, indicating unilateral symptoms [3].

Trends observed for accuracy were mirrored by results for precision. In this study, ICC confidence intervals (95%) for Fitbit Charge 2 HR and Garmin Vivosmart 3 were −0.57 to 0.74 and 0.96 to 0.99, respectively. These matched the ICC confidence intervals reported by Wendel et al [2] for Fitbit Surge and Zip (95% CI 0.06-0.64 and CI 0.96-0.99, respectively). Lamont et al [3] reported ICC confidence intervals for Fitbit Charge HR and Garmin Vivosmart HR of 0.76 to 0.94 and 0.85 to 0.97, respectively. Together, these findings demonstrate that motion sensors, particularly waist-worn devices, can be used to accurately and precisely record steps during overground walking. However, deviations in accuracy and precision may be influenced by PD-related symptoms or gait disturbances.

Our results further support the use of Fitbit and Garmin motion sensors for detecting steps at a CWS and provide evidence demonstrating the usefulness of these devices in the context of the treadmill and longer walking bouts. The examination of a 6-min walking bout is important, as the first 2 to 3 min of walking typically reflects a mixture of anaerobic and aerobic metabolic processes, and the metabolic processes after this period represent aerobic work (ie, the participant has achieved steady state). This may better reflect prolonged walking in daily life as evidenced in other neurological diseases, such as multiple sclerosis [4]. Moreover, the findings of this study further support the use of hip-worn sensors as reported by Wendel et al [2]. This is critical because the commercial availability of waist-worn motion sensors is now rather limited (Fitbit has even discontinued production of Fitbit One), whereas wrist-worn sensors have surged in popularity over recent years.

In summary, there are studies that support the use of a variety of consumer motion sensors for detecting steps in PD. On the basis of the findings of this study and those reported previously [2,3], waist-worn and certain wrist-worn motion sensors can provide accurate and precise records of steps during overground walking. Nevertheless, people with PD might undertake treadmill walking for home-based physical activity, and handrail use is common. These results suggest that the wrist-worn devices would not be ideal for self-monitoring physical activity in this context.

### Study Limitations

Walking was performed under controlled conditions that may not resemble real-world factors that can influence walking, such as the terrain, obstacles, and weather. This study examined single bouts of overground and treadmill walking at a CWS versus examining step counts at various speeds (eg, slow, normal, and fast). Actual steps were manually counted by only 1 research staff member, and this might have introduced error into the gold standard measure of steps taken. Participants were ambulatory and physically independent. The findings may not be generalizable to people with PD at higher disability levels who have balance deficits and/or use assistive devices, such as canes and walkers.

## Abbreviations

CWS: comfortable walking speed
ICC: intraclass correlation coefficient
MDS-UPDRS: Movement Disorder Society version of the Unified Parkinson’s Disease Rating Scale
NIH: National Institutes of Health
PD: Parkinson disease
